# Tranexamic Acid for the Emergency Treatment of Angiotensin-Converting Enzyme Inhibitor-Induced Angioedema

**DOI:** 10.7759/cureus.18116

**Published:** 2021-09-20

**Authors:** Shannon Hasara, Kayla Wilson, John Amatea, Jonathan Anderson

**Affiliations:** 1 Department of Pharmacy, Lakeland Regional Health, Lakeland, USA; 2 Department of Emergency Medicine, Lakeland Regional Health, Lakeland, USA

**Keywords:** angioedema, tranexamic acid, bradykinin, angiotensin converting enzyme inhibitors, drug-induced angioedema

## Abstract

Introduction: Angioedema is a rare but potentially life-threatening adverse effect associated with the use of angiotensin-converting enzyme (ACE) inhibitors. Various therapies, including ecallantide, icatibant, complement-1 esterase inhibitors, and fresh frozen plasma, have been used for treatment with inconsistent results and significant adverse effects. Tranexamic acid (TXA) is used as an alternative for the treatment of hereditary angioedema and it may be an attractive option for the treatment of ACE inhibitor-induced angioedema (ACEi-AE) in the emergency department (ED). The purpose of this study was to evaluate the impact of TXA administration on rates of intubation in patients presenting to the ED with suspected ACEi-AE.

Methods: This was an institutional review board-approved, retrospective cohort study conducted at a single-site ED. All patients who received TXA for ACEi-AE in the ED between January 1, 2019 and March 31, 2021 were eligible for inclusion. The primary outcome was the proportion of patients who required intubation for suspected ACEi-AE.

Results: A total of 16 patients received TXA in the ED for suspected ACEi-AE during the study timeframe. Of these, two patients were intubated prior to administration of TXA. The remaining 14 patients did not require intubation following TXA administration.

Conclusion: Administration of TXA was associated with a low rate of adverse effects and did not contribute to further morbidity when added to standard care in patients presenting to the ED with suspected ACEi-AE.

## Introduction

Angioedema results in more than 100,000 emergency department (ED) visits in the United States (US) each year with approximately 11% of patients requiring admission [1]. This transient, non-pitting edema can affect various sites and may progress to life-threatening airway edema requiring intubation if not expeditiously treated. In most patients, the cause of angioedema is unknown; however, the pathophysiology can be attributed to either histamine- or bradykinin-mediated mechanisms. Histamine-mediated angioedema is the result of mast cell degranulation in response to a type I immunoglobulin E (IgE) hypersensitivity reaction and accounts for 40% to 70% of all cases. While bradykinin-mediated angioedema is less common, these cases may be persistent and more severe due to significant upper airway involvement. Bradykinin-mediated angioedema can be further classified as hereditary angioedema (HAE), acquired angioedema, or angiotensin-converting enzyme (ACE) inhibitor-induced angioedema (ACEi-AE) [2].

ACE inhibitors are listed in the top five most commonly prescribed medications in the US among adult patients aged 40 to 79 years [3]. Inhibition of ACE results in an accumulation of bradykinin, a vasoactive peptide that alters vascular permeability and leads to swelling. ACEi-AE is a rare adverse effect associated with the use of ACE inhibitors affecting 0.1% to 0.7% of treated patients, which accounts for 30% of all angioedema cases [2,4]. Factors associated with a higher incidence of ACEi-AE include female gender, African American ethnicity, heart failure diagnosis, and concomitant therapy with immunosuppressants or dipeptidyl peptidase-IV (DPP-4) inhibitors [2,5]. The risk of ACEi-AE is not dose-related and is highest within the first 30 days; however, it can occur at any point during the course of therapy and may recur up to 72 months after drug discontinuation [2,6,7].

Initial management of ACEi-AE centers on airway management and treating the underlying cause; however, no Food and Drug Administration (FDA) approved therapies or pharmacologic standards of care have been established for this condition. Administration of histamine-1 and histamine-2 receptor antagonists, corticosteroids, and intramuscular (IM) epinephrine may be considered to help differentiate between histamine- and bradykinin-mediated angioedema as they are unlikely to be beneficial in the latter [2]. Other bradykinin-targeted therapies, such as ecallantide and icatibant, have been utilized in patients with ACEi-AE and failed to consistently demonstrate clinical efficacy [8-11]. Complement-1 esterase inhibitors (C1-INH), which are FDA approved for HAE attacks, have demonstrated improvements in time to symptom resolution in various case reports; however, the cost of these medications may prevent their inclusion on institutional formularies [12]. Fresh frozen plasma (FFP) may be used to replete ACE and C1-INH and is less cost-prohibitive than other targeted therapies. FFP carries a risk of hypersensitivity reactions, infection transmission, volume-associated circulatory injuries, and worsening angioedema symptoms [6,13].

Tranexamic acid (TXA) is used as an alternative therapy for long-term prophylaxis in HAE by inhibiting the activation of plasminogen to plasmin and thus decreasing bradykinin production. The role of TXA in the acute treatment of bradykinin-mediated angioedema is less clear. Beauchêne and colleagues conducted a retrospective analysis of 33 patients who received TXA for severe ACEi-AE and noted significant improvements in symptoms with TXA alone in 27 patients (82%) as well as avoidance of intubation in all patients not intubated prior to administration (n = 31) [14]. TXA is generally well tolerated, inexpensive, and easy to administer, making it an attractive option for treating patients with ACEi-AE in the ED; however, documentation of its use in this setting is limited to a single case report to date [15].

The purpose of this study is to evaluate the impact of TXA administration on rates of intubation in patients presenting to the ED with suspected ACEi-AE.

## Materials and methods

This was a single-center, institutional review board-approved (Lakeland Regional Health Institutional Review Board: approval #1760586-1), retrospective study conducted in a 165-bed ED with a pre-pandemic volume of more than 210,000 annual patient visits. At the study institution, there is not a specific protocol for the treatment of suspected or confirmed ACEi-AE; therefore, the use of TXA is at the discretion of the physician. Ecallantide, icatibant, and C1-INH concentrates are not available on the institutional formulary.

An analytics report identified all patients who received TXA for ACEi-AE in the ED between January 1, 2019 and March 31, 2021. Patients were screened in reverse chronological order and included if they were greater than or equal to 18 years of age with recent ACE inhibitor use per physician documentation or outpatient fill history. Recent ACE inhibitor use was defined as patient-reported administration or an outpatient fill (as noted in the external prescription history) within the last 90 days. This timeframe was selected to capture those patients actively receiving treatment with an ACE inhibitor. Patients with signs and symptoms consistent with histamine-mediated angioedema or anaphylaxis; family history of hereditary or acquired angioedema; administration of icatibant, ecallantide, or a C1-INH product prior to receiving TXA; or admission to the trauma service were excluded.

The primary outcome of the study was the proportion of patients who required intubation for suspected ACEi-AE following TXA administration. The secondary outcomes were the rate of hospital admission from the ED, time to resolution of symptoms (per ED provider re-examination documentation), the incidence of adverse effects (including hypotension, anaphylaxis, thrombosis within 28 days, volume-associated circulatory injuries, and worsening angioedema), and ED length of stay (LOS). Descriptive statistics were utilized to report all outcomes.

## Results

A total of 16 patients were treated with TXA in the ED for suspected ACEi-AE during the study time period (Table 1). The majority of patients were Caucasian and male with an average age of 64 years. Lisinopril was the agent most frequently implicated in the development of ACEi-AE with symptoms presenting at a median of 22 months after initiation of therapy. Additional concomitant therapies included non-steroidal anti-inflammatory drugs and calcium channel blockers. All patients presented with facial swelling, and the majority experienced edema localized to the tongue. The median time from symptom onset to ED presentation was 246 minutes. At least one histamine-targeted therapy was administered in the ED to all patients except one. This patient was expeditiously intubated upon arrival and transferred to the intensive care unit (ICU) where histamine-mediated therapies were administered on a scheduled basis. All study patients, with the exception of one, received TXA 1,000 mg IV administered over 10 minutes with a median time to administration of 269 minutes and 41 minutes from the onset of symptoms and ED presentation, respectively. One patient received TXA 100 mg IV. Four patients received two units of FFP in addition to TXA.

**Table 1:** Patient characteristics. Data expressed as n (%) unless otherwise specified. SD: standard deviation; DPP: dipeptidyl peptidase; ACE: angiotensin-converting enzyme; IQR: interquartile range; ED: emergency department; IM: intramuscular; SC: subcutaneous; TXA: tranexamic acid; FFP: fresh frozen plasma.

	Total (n = 16)
Age (years), mean (SD)	64.3 (8.6)
Gender (male)	10 (62.5)
Ethnicity	
African American	5 (31)
Hispanic	2 (13)
White	9 (56)
Other	0 (0)
Past medical history	
Venous thromboembolism	0 (0)
Congestive heart failure	1 (6)
Concomitant therapies	
DPP-IV inhibitor	0 (0)
Calcium channel blocker	4 (25)
Immunosuppressive therapy	0 (0)
Tissue plasminogen activator	0 (0)
Aliskiren	0 (0)
Non-steroidal anti-inflammatory drug	7 (44)
Estrogen-containing product	0 (0)
Outpatient ACE inhibitor	
Lisinopril	12 (75)
Lisinopril/hydrochlorothiazide	1 (6)
Amlodipine/benazepril	3 (19)
Duration of ACE inhibitor use (months), median (IQR)	22.5 (9 - 54.5)
Clinical presentation	
Erythema	0 (0)
Dyspnea	4 (25)
Abdominal pain	0 (0)
Peripheral swelling	0 (0)
Facial swelling	16 (100)
Facial swelling distribution	
Tongue	11 (69)
Lips	5 (31)
Onset of symptoms to ED presentation (minutes), median (IQR)	246 (150 - 697)
Medication administration	
Diphenhydramine	13 (81)
Famotidine	10 (63)
Methylprednisolone	12 (75)
Nebulized racemic epinephrine	3 (19)
IM epinephrine	1 (6)
SC epinephrine	1 (6)
TXA	16 (100)
FFP	4 (25)

No adverse events were documented following the administration of TXA (Table 2). Over 85% of patients experienced partial or complete resolution of symptoms with only three patients admitted to the ICU for a higher level of care. The remaining patients were admitted to the medical floor for observation or discharged home. The average ED LOS was approximately 3.5 hours. Of the four patients presenting with dyspnea, two required intubation prior to administration of TXA. Intubation was avoided in the remaining 14 patients. Ultimately, all patients survived to ED or hospital discharge, and only one patient experienced a recurrent attack within 28 days necessitating another ED visit.

**Table 2:** Clinical outcomes. Data expressed as n (%) unless otherwise specified. TXA: tranexamic acid; FFP: fresh frozen plasma; ED: emergency department; ICU: intensive care unit; LOS: length of stay; SD: standard deviation; IQR: interquartile range.

	Total (n = 16)
TXA adverse effects	
Hypotension	0 (0)
Anaphylaxis or hypersensitivity reaction	0 (0)
Thrombosis within 28 days	0 (0)
FFP adverse effects	
Transfusion-associated circulatory overload	0 (0)
Transfusion-related acute lung injury	0 (0)
Worsening angioedema	0 (0)
Resolution of symptoms	
None	2 (13)
Partial	12 (74)
Complete	2 (13)
Time from TXA administration to resolution (minutes), mean (SD)	100 (26)
Repeat TXA dose required	1 (6)
Repeat FFP dose required	0 (0)
ED disposition	
Home	6 (38)
Medical floor	7 (44)
ICU	3 (18)
ED LOS (hours), mean (SD)	3.5 (1.9)
Mechanical ventilation required	2 (13)
Duration of mechanical ventilation (days), mean (SD)	2.5 (1.4)
Hospital LOS (days), median (IQR)	1.5 (0.8 - 2.8)
ICU transfer required	3 (18)
ICU LOS (days), median (IQR)	1.9 (1.5 - 6.2)
Discharge disposition	
Alive	16 (100)
Deceased	0 (0)
Palliative	0 (0)
ED visit for recurrent attack within 90 days	1 (6)

## Discussion

Few studies have evaluated the use of TXA for the treatment of ACEi-AE. The first report, published by Beauchêne et al., was a retrospective analysis of 33 patients treated with TXA for ACEi-AE within the ED of two French hospitals [14]. Patients received IV or oral (PO) TXA at dosages ranging from 500 mg to 4,000 mg. Two patients required intubation prior to administration of TXA. Intubation was avoided in the remaining patients, and all patients survived to discharge. The majority of patients experienced at least a partial resolution of symptoms; however, six patients did require subsequent treatment with icatibant or C1-INH concentrate. The second was a case report from Wang and colleagues describing the complete resolution of ACEi-AE following the administration of TXA in the ED [15]. In addition to histamine-targeted therapies, the patient received TXA 1,000 mg IV over 10 minutes, and experienced resolution of symptoms within two hours. The patient was discharged from the ED without any complications, adverse effects, or need for further intervention.

Based on the paucity of data, there is no consensus on the role of TXA in treating ACEi-AE; however, the results of this study, together with those from the Beauchêne and Wang studies, suggest that the use of TXA for the treatment of angioedema in the ED is safe and does not contribute to further morbidity. Treatment with TXA was well-tolerated with no reports of hypotension, anaphylactic or hypersensitivity reactions, or thrombosis within 28 days. It is important to note that none of the patients in this study had an active thromboembolic disease, which would have contraindicated the use of TXA. Only two patients in this study required mechanical ventilation, both of whom were intubated prior to administration of TXA. Intubation was avoided in the remaining 14 patients; however, the frequency of intubation associated with ACEi-AE is generally low. A retrospective study by Mudd and colleagues demonstrated an intubation rate of 9.5% in this population [16]. This was corroborated by Keh et al. who found that 3 of 32 patients (9.4%) presenting to the ED with ACEi-AE required intubation [17].

This study included one patient who received a repeat dose of TXA, which, to the authors’ knowledge, has not been previously reported in the literature for the acute treatment of ACEi-AE. The patient was a 63-year-old male who presented to the ED with recurrent tongue swelling that began approximately three hours prior to arrival. Of note, this was the fifth episode of angioedema he had experienced within the last four months after over four years of treatment with amlodipine-benazepril. Following the first ED visit, his amlodipine-benazepril was changed to amlodipine monotherapy. Amlodipine was continued despite subsequent episodes. During the third ED visit, he received TXA 1,000 mg IV over 10 minutes and was noted to have a partial resolution of his symptoms. For the most recent episode, he received diphenhydramine 50 mg IV, methylprednisolone 125 mg IV, nebulized racemic epinephrine, and TXA 1,000 mg IV over 10 minutes. Upon re-evaluation, his symptoms appeared to be slowly improving. He was able to swallow but his tongue remained swollen and his voice was still muffled. A repeat dose of TXA 1,000 mg IV over 10 minutes was administered, and the patient was admitted for observation. The following day, the patient’s facial swelling was noted to have resolved. No complications or adverse effects were observed, and the patient was subsequently discharged.

There are several limitations that should be considered when evaluating the results of this study. As a retrospective study, all of the results rely on the availability and accuracy of the data within the electronic health record. This includes clinical outcomes data such as thrombosis within 28 days or ED visits for recurrent attack within 90 days as this information will only be available for patients presenting to this institution. It does not account for patients seen at other institutions for these problems. The duration of symptoms prior to ED presentation as well as the time to and extent of symptom resolution was documented in less than half of treated patients. This study was conducted within a single-site ED, which may limit generalizability to other institutions with dissimilar protocols and processes. Additionally, histamine-targeted therapies may be used to differentiate between histamine- and bradykinin-mediated angioedema; however, these medications were often administered in conjunction with TXA, making it difficult to determine whether these agents contributed to symptom resolution or prevention of adverse effects from TXA. Finally, the diagnosis of ACEi-AE relies on clinical presentation and a history of ACE inhibitor use as there are no confirmatory diagnostic tests available.

## Conclusions

This study suggests that the administration of TXA in addition to standard care was associated with a low rate of adverse effects in patients presenting to the ED with suspected ACEi-AE. These findings are consistent with other published data and may support the incorporation of TXA into ED angioedema treatment plans; however, larger studies are needed to confirm its place in therapy.
